# Piezoelectric PVDF Nanoparticles for Enhanced Antimicrobial Activity via Mechanical Stimulation: A Proof-of-Concept Study

**DOI:** 10.1021/acsami.4c22219

**Published:** 2025-02-24

**Authors:** Mariana P. Fernandes, Joana Moreira, Marta Fernandes, Andrea Zille, Carla Silva, Filipe Samuel Silva, Margarida M. Fernandes

**Affiliations:** † Center for Micro-Electro Mechanical Systems, 56059 University of Minho, Campus Azurém, Guimarães 4800-058, Portugal; ‡ LABBELS-Associate Laboratory in Biotechnology and Bioengineering and Microelectromechanical Systems, University of Minho, 4710-057 Braga, Portugal; § Centre of Chemistry, University of Minho, Campus Gualtar, Braga 4710-057, Portugal; ∥ Centre of Physics, University of Minho, Campus Gualtar, Braga 4710-057, Portugal; ⊥ 2C2T—Centre for Textile Science and Technology, University of Minho, Campus Azurém, Guimarães 4800-058, Portugal; # Centre of Biological Engineering, University of Minho, Campus Gualtar, Braga 4710-057, Portugal

**Keywords:** piezoelectricity, PVDF, nanoparticles, antimicrobial, mechanical stimulation

## Abstract

The alarming rise of antimicrobial resistance is a public health issue, driven by the excessive and improper use of antibiotics, which are becoming less effective against an increasing number of microorganisms. There is an urgent need to find alternative antimicrobial strategies that can bypass bacterial resistance mechanisms. Using physical stimuli to sensitize bacteria to antimicrobial action is one step toward addressing this challenge. In this work, piezoelectric poly­(vinylidene fluoride) (PVDF) nanoparticles were developed in an attempt to control and enhance the antimicrobial activity of materials through piezoelectric stimulation. The nanoparticles exhibited sizes ranging from 200 to 400 nm, with low polydispersity, a negative surface charge, and a spherical and smooth morphology. Using the reprecipitation methodology, the nanoparticles were synthesized through the crystallization of PVDF in the electroactive β-phase, achieving percentages of formulations greater than 80%. These nanoparticles demonstrated promising antimicrobial properties, which were considerably enhanced through dynamic conditions involving mechanical stimulation resulting in the creation of electroactive microenvironments. Notably, this dynamic approach exhibited a stronger inhibitory effect on bacterial growth, particularly against *Escherichia coli*. When water was used as nonsolvent for increasing the PVDF concentration to 10 mg/mL, it resulted in greater bacterial inhibition, with reductions of 1.33 log_10_ under static conditions and 2.21 log_10_ under dynamic conditions. However, this effect is less pronounced for *Staphylococcus aureus*. In contrast, when 50% ethanol solution is used as nonsolvent, both bacteria exhibited significant reductions: *E. coli* was completely eradicated under static conditions, while *S. aureus* showed a 1.93 log_10_ reduction. Under dynamic conditions, both bacteria were completely eliminated. Although these nanoparticles compromise the viability of human fibroblasts after 72 h of contact, this study provides a proof-of-concept for materials that enhance antimicrobial activity through mechanical stimulation. These findings open possibilities for developing hygienic coatings on public surfaces, leveraging pressure or touch to activate antibacterial effects.

## Graphical abstract

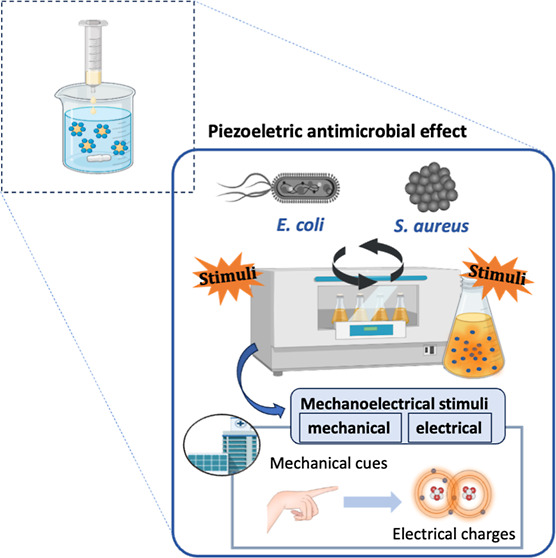

## 1 Introduction

The incidence of infections caused by antibiotic-resistant bacteria is a growing concern today, as it poses significant challenges to public health, medical treatment, and healthcare systems. Hospital infections caused by microorganisms such as *Staphylococcus aureus* and *Escherichia coli* have been responsible for 110,000 deaths per year in all European Union (EU) countries, in part due to the increased resistance of these microorganisms.^1,2^ Although these Gram-positive and Gram-negative bacteria have conserved structural membranes, chemical modifications in lipids and cell wall components give them resistance to antibiotics that interact with the membrane function. In addition to this, bacteria have developed mechanisms such as efflux pumps, proteolytic degradation and secretion of factors that neutralize these antibiotics.^3^ Therefore, the development of new therapeutic approaches has been extensively studied.

The development of advanced antibacterial strategies has increasingly focused on stimulus-responsive materials, such as piezoelectric and photocatalytic systems, to combat microbial resistance. The use of electroactive polymers, mainly based on poly­(vinylidene fluoride), PVDF, with an emphasis on the development of advanced multifunctional materials, is a strategy that is gaining a huge attention, since the sensibility of eukaryotic and prokaryotic cells to detect a physical stimuli and translate them into biological and biochemical responses has already been proven.^4^ Many studies have already reported the development of PVDF-based materials for antimicrobial approaches in the form of nanocomposites, 2D films, and 3D structures.^1,2,5,6^ For these applications, it is important to ensure that an electrically active polymorph PVDF is obtained, i.e., crystallization occurs in one of the electroactive phases. When PVDF is crystallized in one of the electroactive phases, that is, in the β phase, it may result in a material with a high piezoelectric coefficient (|*d*_33_| = 34 pC·N^–1^), due to the chain conformation that leads to the highest dipole moment per unit cell. This conformation can be achieved by crystallizing the polymer at room temperature.^7^ In addition, the use of piezoelectric materials, which generate electrical charges in response to mechanical stimuli, such as pressure, has emerged as a need for a transformative tool for several applications, namely, antibacterial,^8^ but also in the biomedical field.^9^ These materials may provide a unique way to address critical challenges ranging from antibacterial strategies, tissue regeneration, and anticancer therapies. By harnessing their ability to convert mechanical energy into electrical signals, piezoelectric materials unlock innovative solutions and inspire the development of new treatment applications based on physical stimuli, thus avoiding traditional chemical approaches.^8,9^ For example, Jiang et al. highlight the effectiveness of pressure-induced amorphous imidazole zeolitic structures in anticancer therapies, emphasizing how manipulating materials under pressure can lead to remarkable advances in medicine.^10^

In contrast to piezoelectric materials, photocatalytic materials have also demonstrated significant antibacterial potential. While piezoelectric materials exert mechanical disruption on biofilm structures and membrane depolarization on bacteria, photocatalytic systems degrade the extracellular matrix through the generation of oxidative stress. Upon exposure to light, photocatalytic materials produce reactive oxygen species (ROS), which induce oxidative damage to bacterial cells. For instance, graphene oxide and cuprous oxide (GO–Cu_2_O) nanocomposites have been shown to possess effective bactericidal properties, owing to their photocatalytic capabilities.^11^ However, a key limitation of photocatalysis is its dependence on light irradiation, which restricts its application in low-light environments. In contrast, piezoelectric materials can be activated by mechanical stimuli, providing a greater operational flexibility. Moreover, photocatalytic methods may pose higher risks of cytotoxicity due to the generation of ROS, whereas piezoelectric effects, which are localized to the material’s surface, offer potentially safer alternatives, particularly in biomedical applications.^12^

Nanotechnology is another strategy that is gaining attention as a potential new tool to overcome microbial resistance. Due to its vast encapsulation capacity, it is recurrently used to direct drugs to the site of infection in a controlled manner so that higher doses of the drug can be administered directly to the infected site, thus overcoming resistance and consequently with fewer adverse effects. Furthermore, the nanometric conformation itself has unique properties, different from those of free molecules and bulk materials with the same composition. In general, nanoparticles as microbial resistance moderating agents have been able to overcome mechanisms including decreasing absorption and increasing drug efflux from the bacterial cell, biofilm formation, and intracellular bacteria.^13–15^ This idea of transforming antimicrobial agents themselves into nanometric entities with intrinsically antimicrobial properties has already been described in the literature, notably through the reprecipitation method.^16,17^ However, the design and synthesis of nanoparticles require strict control over their physicochemical properties to achieve the desired functionalities. One of the main challenges lies in the transition from laboratory synthesis methods to industrial-scale processes, which often present significant limitations. To realize the full potential of nanotechnology, it is essential to shift the focus from nanoscale innovations to the development of viable approaches for gram-scale or industrial-scale nanoparticle production.^18^ For example, Zhang et al. highlight the feasibility of large-scale synthesis using oxides as model systems. In this context, they also demonstrated an efficient method for the large-scale synthesis of lanthanum coordination polymers, highlighting their potential in antibacterial and antitumor applications.^19^

Therefore, the possibility of combining both the effect of nanotechnology and that of electrically active microenvironments using the PVDF polymer is an interesting strategy to obtain a synergistic effect for improved antimicrobial effects. This combination can overcome the limitations of traditional chemical-based methods and enhance the intrinsic benefits of nanotechnology in advanced systems to combat microbial resistance.

Although the specific mechanisms by which nanoparticles interact with bacteria are not fully understood, nanoparticles are believed to increase the permeability of the microbial cell membrane, affect the osmotic system and cause cytoplasmic components to flow out of the cell, ultimately resulting in cell death. However, factors such as size, shape, and surface charge impact nanoparticle–cell interactions.^13,20^

As bacterial membrane is extremely evolutionarily conserved, it is unlikely to be altered by a single genetic mutation; this form of action reduces the probability of new resistant strains appearing. Furthermore, nanoparticle systems can target diverse biological pathways, and bacteria must undergo numerous simultaneous changes to become resistant.^14,20^

This work proposes the development of nanoparticles composed of the highly piezoelectric polymer PVDF. The synergistic effect of the antimicrobial agent and the electroactive material in the presence of mechanical stimulus was thus evaluated. Different effects of nanoparticles on Gram-negative and Gram-positive bacteria were studied as follows: (i) under static conditions, i.e., without mechanical stimulation (incubation without rotations) and keeping in mind the inherent effect of the nanoparticles themselves and (ii) under dynamic conditions, i.e., evaluating the mechanoelectric effect, due to incubation at 200 rpm. We hypothesized that nanospherization of this polymer by reprecipitation would extend its activity to Gram-negative and Gram-positive bacteria, in accordance with its bacterial membrane disruption effect. This work thus reports the development of electroactive nanoparticles that exhibit greater antimicrobial activity when a mechanical stimulus is applied, based on the piezoelectric response of materials, especially Gram-negative ones. This strategy represents an advancement in the development of microbial-free coatings that respond to a mechanical stimulus such as vibration, pressure, or touch, effectively inhibiting the proliferation of bacteria. Incorporating these coatings into composites could enhance their antimicrobial performance by taking advantage of their quantum effect.

## 2 Materials and Methods

### 2.1 Synthesis of PVDF Nanoparticles

PVDF nanostructures were produced by a self-assembly method at room temperature, according to the set present in Figure 1. First, PVDF powder was dissolved at concentrations of 5 and 10 mg/mL in *N*,*N*-dimethylformamide (DMF). Then, ethanol and water were combined in two water/ethanol volume ratios (100% water and 50% ethanol) to serve as nonsolvent PVDF mixtures. Under vigorous stirring (500 rpm), 4 mL of PVDF/DMF solution was injected dropwise through gravity using a 20G needle attached to a plastic syrinx into a volume of 16 mL of water or water/ethanol mixture. After injection, stirring was maintained for 10 min at room temperature. After keeping overnight, the precipitate was stored at room temperature or collected by centrifugation for 30 min at 4500 rpm, washed twice with water, and dried at 40 °C.

**Figure 1:**
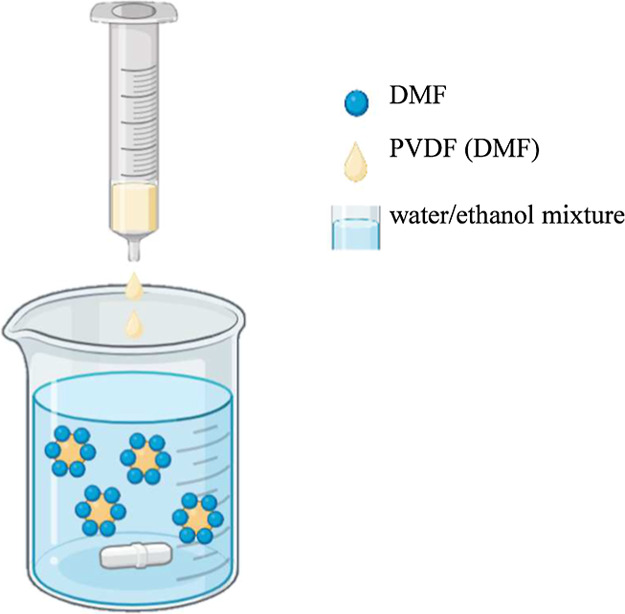
Schematic illustration of the self-assembly method to produce PVDF nanoparticles.

### 2.2 Characterization of PVDF Particles

#### 2.2.1 Physicochemical Characterization of the PVDF Particles

The mean size diameter, polydispersity index (PDI), and surface charge (Z-potential) of the PVDF particles were measured using a Zetasizer Nano ZS (Malvern Instruments) at 25 °C. The samples were read in triplicate, the results being described as mean ± standard deviation.

#### 2.2.2 Scanning Electron Microscopy

The PVDF particles were added onto aluminum stubs using conductive carbon adhesive tape and coated with a thin layer of Au for 90 s (approximately 7.5 nm) with a sputter coater Quorum Mini QS (London, England). Scanning electron microscopy (SEM) images were taken using a FlexSEM 1000 (Hitachi, Japan) scanning electron microscope at a voltage of 10 kV.

#### 2.2.3 Fourier Transform Infrared Spectroscopy

Fourier Transform Infrared Spectroscopy (FTIR) was used to analyze the chemical structure of the PVDF particles. The spectra were obtained using a Bruker Alpha II instrument (Massachusetts, USA) using Opus 8.22.28 software. Samples were placed directly on the crystal, and spectra were acquired between 400 and 4000 cm^–1^ wavenumbers at a resolution of 2 cm^–1^.

Assuming that the FTIR absorption follows the Lambert–Beer’s Law, the absorption coefficients, Kα and Kβ, were calculated at the respective wavenumber of 766 and 840 cm^–1^. In this way, the relative fraction of the β-phase in a sample containing α and β phases was calculated by eq 1

1 $$F\left(\beta \right)=\frac{A_{\beta }}{\left(\frac{K_{\beta }}{K_{\alpha }}\right)A_{\alpha }+A_{\beta }}$$

where *F*(β) represents the β-phase content; *A*_α_ and *A*_β_ the absorbance at 766 and 840 cm^–1^; *K*_α_ and *K*_β_ are the absorption coefficients at the respective wavenumbers, whose values are 6.1 × 10^4^ and 7.7 × 10^4^ cm^2^ mol^–1^, respectively.^5^

#### 2.2.4 Simultaneous Thermogravimetric Analysis

TGA was performed on a Hitachi STA 7200 (TA Instruments, Newcastle, UK). The PVDF particles weighing about 3–7 mg were heated in hermetically sealed aluminum pans and tested in the temperature range of 30–600 °C at a heating rate of 10 °C·min^–1^, under an inert nitrogen atmosphere at 200 mL·min^–1^ flow rate.

The degree of crystallinity (Δ*X*_c_) of the nanoparticles was determined using eq 2

2 $$\Delta X_{c}=\frac{\Delta H}{x\Delta H_{\alpha }+y\Delta H_{\beta }}$$

where Δ*H* is the melting enthalpy of the sample and Δ*H*_α_ (93.07 J·g^–1^) and Δ*H*_β_ (103.4 J·g^–1^) are the enthalpies of a 100% crystalline sample in the α- and β-phases, respectively. Further, *x* and *y* represent the α- and β-phase content of the nanoparticles, obtained from the FTIR measurements.^5^

### 2.3 Antimicrobial Activity

Two different methods were used to evaluate the antimicrobial properties of PVDF nanoparticles: the quantitative analysis by Colony Forming Unit (CFU) after contact with the nanoparticles and the Live/Dead kit that qualitatively evaluates the viable and nonviable bacteria in suspension in contact with nanoparticles.

The antimicrobial activity of the samples was evaluated against Gram-positive *S. aureus* and Gram-negative *E. coli*. The bacteria were cultured in Nutrient Broth (NB, Merck, Darmastadt, Germany) overnight at 37 °C with aeration, and the next morning, the cultures were moved to a fresh medium with an initial optical density of 0.1 (at 600 nm). The bacterial suspensions were then allowed to grow for 2 h at 37 °C under both static and dynamic conditions, that is, without rotation and at 200 rpm, respectively (Figure 2). Specifically, 200 μL samples were laid in contact with 200 μL of the inoculum for 2 h. The suspensions obtained were subjected to 8 serial dilutions (1:10) in a sterile 0.9% (w/v) NaCl solution. For each dilution, a volume of 20 μL was dropped on the NB plate agar and further incubated at 37 °C for 24 h. Following, the number of viable bacteria was determined, allowing a quantitative analysis of the average CFU·mL^–1^. The bactericidal activity was evaluated with three independent assays as log_10_ for each condition.^1^

**Figure 2:**
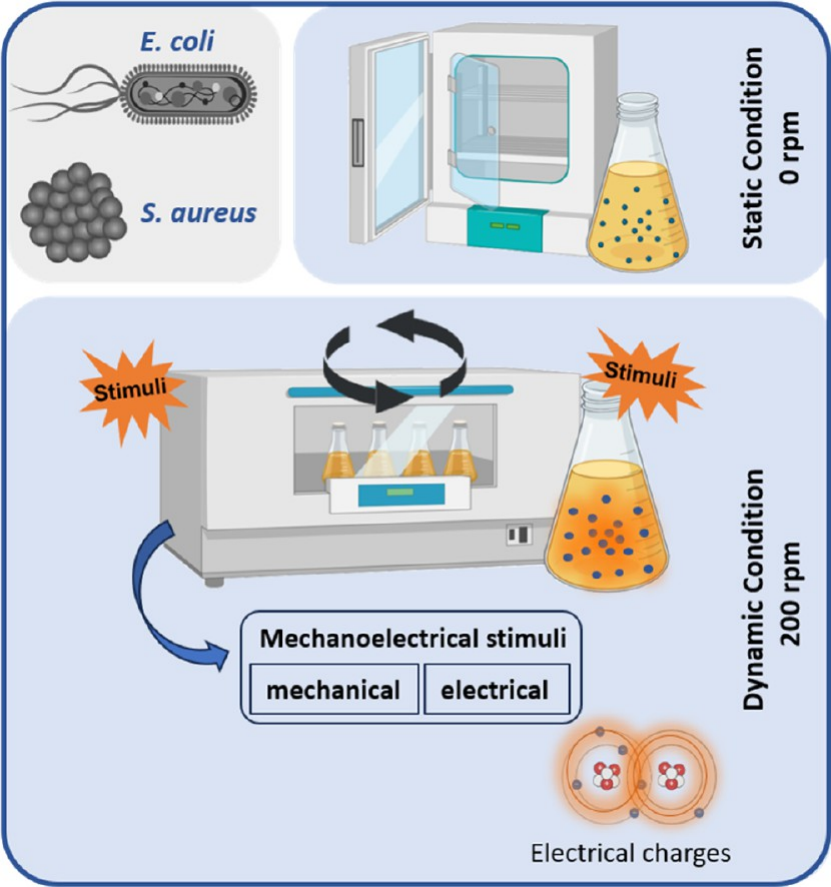
Schematic presentation of the static and dynamic stimulation of the piezoelectric nanoparticles in the antimicrobial tests.

Bacterial viability was assessed by microscopy using the Live/Dead TM BacLightTM Bacterial Viability Kit (Invitrogen, US). After incubation for 2 h at 37 °C under both static and dynamic conditions, 20 μL of the particle’s suspension was stained for 15 min in the absence of light with a mixture of 1.5 μL of green-fluorescent SYTO 9 and red-fluorescent propidium iodide. Finally, the imaging of the samples was performed using a fluorescence microscope (Olympus BX51 microscope). The representative images were taken at magnifications of 10× and 100×.

#### 2.3.1 Scanning Electron Microscopy

The impact of piezoelectricity in nanoparticles on bacterial structural and morphological changes and the interaction between nanoparticles and the bacteria under study were investigated by using SEM (JEOL JSM-6010LV). After the antibacterial assay, the bacteria in contact with the nanoparticle suspension were fixed with 3% (v/v) glutaraldehyde to the glass for 30 min and washed twice with PBS and dehydrated with ethanol [concentration of 30, 50, 70, 80, 90 and 100% (v/v)]. Prior to SEM observation, the samples were coated with gold (gold sputtering equipment: Quorum/Polaron model E6700; accelerating voltage, 5.00 kV).

### 2.4 Cytotoxicity Activity

#### 2.4.1 Cell Culture Maintenance

The BJ-5ta adherent cell was cultured in 4 vol parts of Dulbecco’s Modified Eagle Medium and 1 vol part of medium 199, supplemented with 0.01 mg/mL hygromycin B, 10% (v/v) heat-inactivated Fetal bovine serum, and 1% (v/v) antibiotic/antimycotic mixture. Cells were grown in 75 cm^2^ tissue culture flasks in a humidified environment with 5% CO_2_ at 37 °C. The cell culture medium was renewed twice per week. The adherent cells were detached with a 0.05% (w/v) trypsin solution for subcultures and plating.^21^

#### 2.4.2 Cell Viability Assessed by 3-(4,5-Dimethylthiazol-2-yl)-5-(3-carboxymethoxyphenyl)-2-(4-sulfophenyl)-2*H*-tetrazolium (MTS) Assay

The cell proliferation MTS assay was used to assess the cell viability following exposure to PVDF nanoparticles. BJ-5ta cells were seeded at a density of 10,000 cells/well on a 96-well tissue culture plate, the day before the experiments. Cells were exposed to different concentrations of PVDF particles in aqueous/ethanol suspension through successive 1:2 dilutions (for particles prepared with 5 mg/mL PVDF: 0.0195 0.039, 0.0781, 0.156, 0.312, and 0.625 mg/mL; for particles prepared with 10 mg/mL PVDF: 0.039 0.0781, 0.156, 0.312, and 1.25 mg/mL). The higher concentration values of PVDF in each nanoparticles suspension correspond to the formulation without any dilution. Cells exposed to 3% of DMSO were used as control of death, cells incubated only with culture media were used as life control, and cells incubated with water/50% ethanol at the same dilutions as the particles were used as solvent controls. Cell metabolic activity was measured using the MTS viability assay after 24 and 72 h of contact, according to the manufacturer’s protocol. The reduction in MTS induced by viable cells was measured in a microplate reader by using a 96-well plate reader at 490 nm (Synergy H11, BIOTEK). The relative viability was calculated and represented graphically with respect to the life control. Each sample was assayed in triplicate.^21^

### 2.5 Statistical Analysis

The results are presented as the average of individual measurements with the respective standard deviations and analyzed by the GraphPad Prism 5.0 program (La Jolla, CA, USA). To determine the statistical significance, one-way analysis of variance was used, followed by the unpaired two-tailed Student’s *t*-test method.

## 3 Results and Discussion

### 3.1 PVDF Nanoparticles Synthesis and Physical Stability

The synthesis of PVDF nanoparticles was achieved through a rapid emulsification/reprecipitation process. In this method, PVDF/DMF droplets are uniformly dispersed in the aqueous phase, facilitating solvent exchange between the PVDF–DMF oil droplets and the surrounding water. This exchange triggers the restructuring and shrinkage of the droplets induced by the aqueous phase, resulting in the formation of the nanoparticles.

The physical stability of 5 and 10 mg/mL PVDF particles synthesized using water as nonsolvent was evaluated over 3 weeks through the assessment of their size, polydispersity, and zeta potential. Figure 3 confirms that there was a noticeable trend of the particle sizes ranging from 255 to 270 nm, a PDI of approximately 0.250, and surface charges between −10 and −15 mV over time. In fact, both negative and positive charges may be found on PVDF membranes and films, as described in the literature.^6,7^ This behavior is mostly influenced by the polarization of PVDF, which determines whether fluorine or hydrogen atoms are exposed on the surface of the film. The negative charges are typically associated with the exposure of fluorine atoms, while the positive charges arise when hydrogen atoms are predominant.^7^ Additionally, the preferential adsorption of counterions (e.g., Cl– and OH−) onto hydrophobic regions further contributes to the observed charge characteristics in aqueous environments.^22^ For the PVDF nanoparticles presented in this study, it is reasonable to assume that self-assembly results in fluorine atoms being exposed on the outer layer while hydrogen atoms are concentrated in the inner core. Furthermore, the aqueous-based nanoparticles exhibited stability and uniformity, as indicated by their monodisperse nature. The PVDF concentration had a minimal impact on their physical stability.

**Figure 3:**
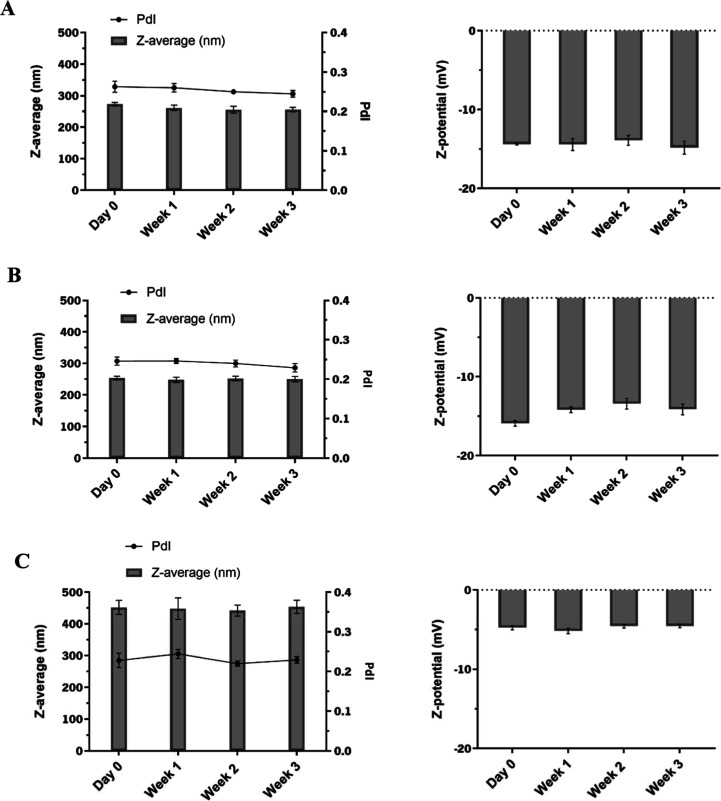
Stability characterization of PVDF particles. The particles were prepared with 5 and 10 mg/mL of PVDF: (A) 5 mg/mL of PVDF with nonsolvent water; (B) 10 mg/mL of PVDF with nonsolvent water; and (C) 5 mg/mL of PVDF with nonsolvent 50% ethanol. The data represent the mean and SD of the *Z*-average (nm), PDI, and surface charge (*Z*-potential (mV)) for 3 weeks of three independent experiments.

Further incorporation of ethanol into the process of nanoparticle development was performed aiming to optimize the synthesis of PVDF nanoparticles. Ethanol may play a critical role in the formation of PVDF nanoparticles. When acting as a nonsolvent for PVDF, ethanol facilitates the polymer crystallization process during the solvent exchange process, which is essential for producing nanoparticles with controlled size and morphology. Its intermediate polarity helps regulate the exchange between DMF (a polar organic solvent) and water (a highly polar nonsolvent), leading to uniform nanoparticles with reduced aggregation.^23^ With this in mind, and considering the previous results, a 50% (v/v H_2_O) ethanol solution was selected as the nonsolvent for the nanoparticles production, using an initial PVDF concentration of 5 mg/mL. The resulting particles exhibited sizes of around 460 nm, a surface charge of approximately −6 mV, and a PDI of 0.250 (Figure 3). These results indicate that the particles are stable and uniform over time and display a monodisperse character. Nevertheless, when comparing nanoparticles synthesized using water to those synthesized with an aqueous ethanolic solution, a noticeable increase in particle size is observed, nearly doubling—a result contrary to the predictions. This phenomenon can be justified by the thermodynamic properties of the PVDF/solvent/nonsolvent system, as well as by the polymer precipitation rate, which depends on mutual mass transfer between three components: the nonsolvent, solvent, and polymer. Therefore, when water is used as the nonsolvent, instantaneous demixing may occur. However, when ethanol is used, delayed demixing occurs, resulting in a lower precipitation rate. This suggests that the diffusion of the nonsolvent and solvent occurs more gradually, leading to slower crystallization. As a result, larger particles or even aggregates may form.^24^

The morphology of PVDF particles was evaluated by SEM (Figure 4).

**Figure 4:**
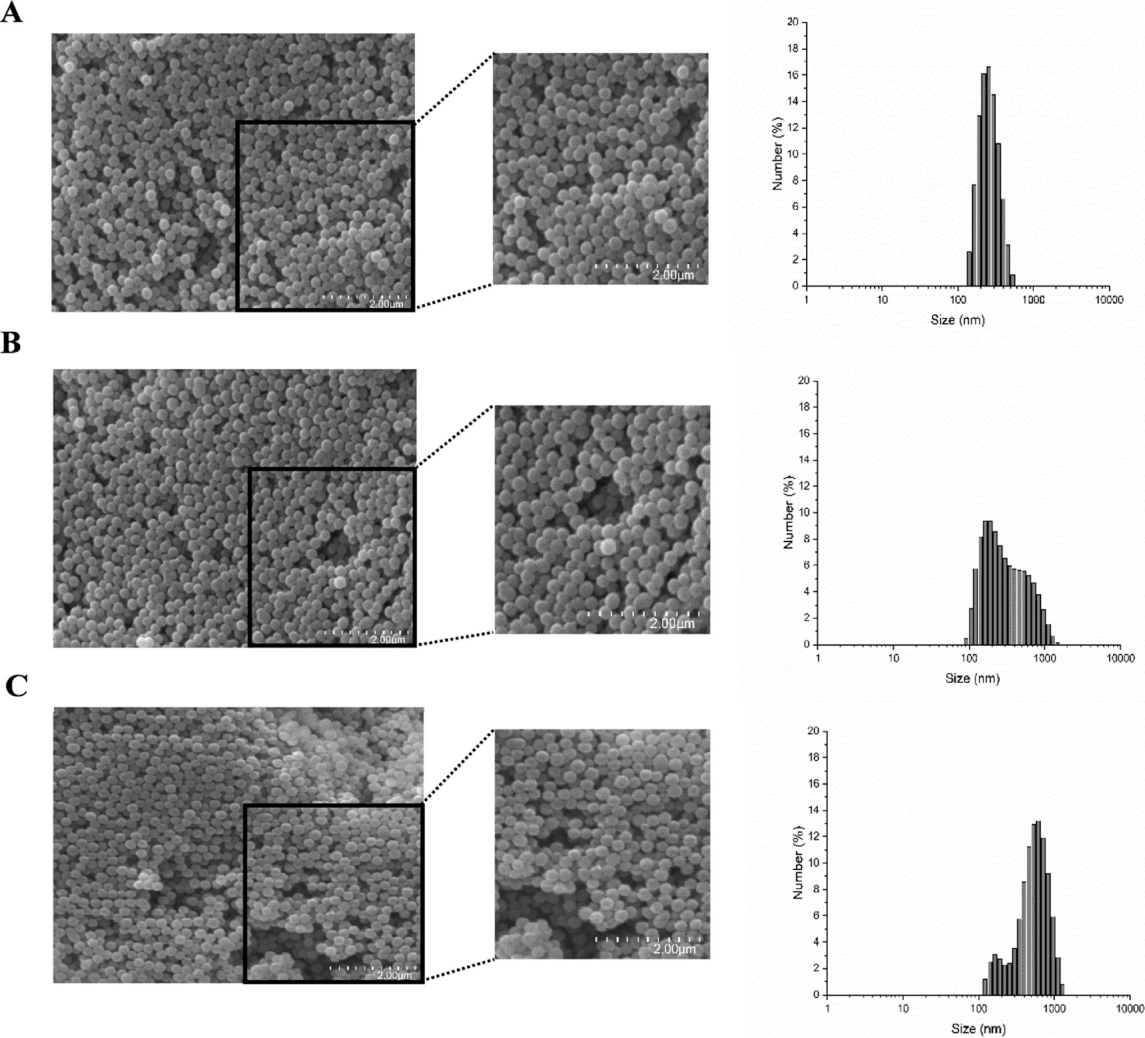
SEM microphotographs (40,000× magnification) and particle size distribution of PVDF particles at different conditions: (A) 5 mg/mL of PVDF with nonsolvent water; (B) 10 mg/mL of PVDF with nonsolvent water; and (C) 5 mg/mL of PVDF with nonsolvent 50% ethanol. The images were amplified from areas of the SEM micrographs for better visualization of the particles.

By analyzing SEM images of PVDF particles, it is possible to confirm the ability of the PVDF polymer to crystallize and form uniform, smooth, and spherical particles, with low polydispersity using a self-assembly reprecipitation methodology.

The distribution results (Figure 4) showed that all particles presented a narrow distribution width centered on the mean particle size, in agreement with Figure 3, which suggests a relatively uniform distribution with a well-defined size range. Still, particles with 5 mg/mL PVDF synthesized using water as a nonsolvent were the ones that presented the most symmetrical distribution, while those with 10 mg/mL PVDF presented slight asymmetry to the right and particles with 5 mg/mL PVDF synthesized using ethanolic solution as nonsolvent a slight asymmetry to the left.

### 3.2 Physical-Chemical Characterization of PVDF Nanoparticles

As described in Section 2.2.4, Fourier transform infrared (FTIR) spectroscopy can clarify the crystalline phases of PVDF efficiently. The FTIR spectra of PVDF nanoparticles are represented in Figure 5A, B, where it is possible to detect peaks related to the α, β, and γ phases.

**Figure 5:**
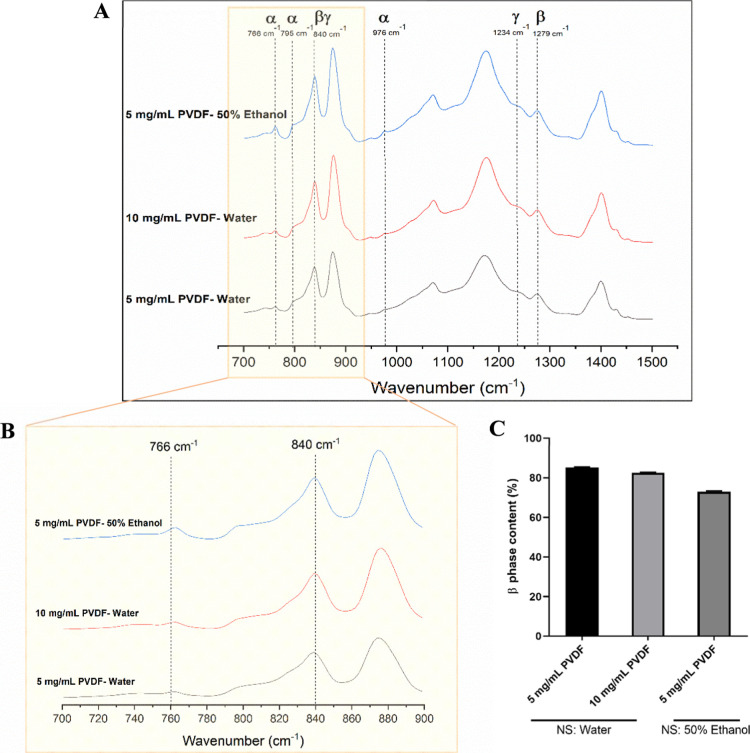
FT-IR spectra from (A) 700 to 1500 cm^–1^ and (B) 700 to 900 cm^–1^ of PVDF nanoparticles prepared by 5 mg/mL PVDF using a 50% ethanol solution as nonsolvent (blue line) and nanoparticles prepared by 5 mg/mL (black line) and 10 mg/mL (red line) of PVDF using water as nonsolvent. (C) β phase content (%) of the PVDF nanoparticles, where NS represents the nonsolvent used.

The peaks at 766, 795, and 976 cm^–1^ are attributed to the nonpolar α phase and are more pronounced when an ethanolic solution is used as the nonsolvent. As shown in Figure 5A,B, the intensity of the α-phase peaks decreases with increasing water content in the nonsolvent, indicating that water exerts an inhibitory effect on the formation of the α-phase during nanoparticle synthesis. On the other hand, the peaks at 1279 and 1234 cm^–1^ are attributed, respectively, to the β and γ phases, and are detected in all particles. Similarly, the intense peak at 840 cm^–1^, originating from a mixture of β and γ phases, is also present in all particles. This peak, along with the α phase peak at 766 cm^–1^, provides the necessary information to quantify the electroactive phase content of PVDF, which can be calculated using eq 1.^5,25^

As shown in Figure 5C, the β-phase percentage values were calculated. For particles synthesized using water as the nonsolvent, the β-phase content was similar, with values of 85% and 82% for PVDF concentrations of 5 and 10 mg/mL, respectively. In contrast, for particles synthesized using a 50% ethanolic solution as the nonsolvent, a more prominent α-phase peak at 766 cm^–1^ was observed, resulting in a lower β-phase content of 73%. Therefore, the content of the electroactive phase increases as the proportion of water in the system rises, due to the strong hydrogen bonding interactions between the solvent (DMF) and the nonsolvent (water), which favor β-phase formation. In contrast, when ethanol is used as a nonsolvent, the weaker interaction between the solvent and nonsolvent allows the PVDF molecules more time to crystallize into the α-phase.^24–26^

The use of water as nonsolvent was found to be more effective for PVDF nanoparticles synthesis, in terms of size, PDI, and electroactive β-phase content of PVDF. The high polarity of water likely facilitates a faster and more efficient solvent exchange compared to ethanol, promoting uniform and compact nucleation. This, in turn, results in smaller particle sizes and a lower PDI. The rapid precipitation induced by water likely favors the kinetic conditions required for the selective formation of the electroactive β-phase. Additionally, the absence of ethanol minimizes potential interference in the crystallization process and eliminates residual effects that could impact the particle size and phase purity. Water’s strong nonsolvent behavior may also enhance hydrophobic interactions, compacting the particle structure and stabilizing the β-phase. These factors collectively make water a more favorable medium for achieving optimal PVDF nanoparticle characteristics.^26–29^

### 3.3 Thermal Characterization

In order to evaluate the thermal properties of the PVDF nanoparticles, TGA, corresponding derivative thermogravimetric (DTG), and differential scanning calorimetry (DSC) measurements were carried out and are presented in Figure 6.

**Figure 6:**
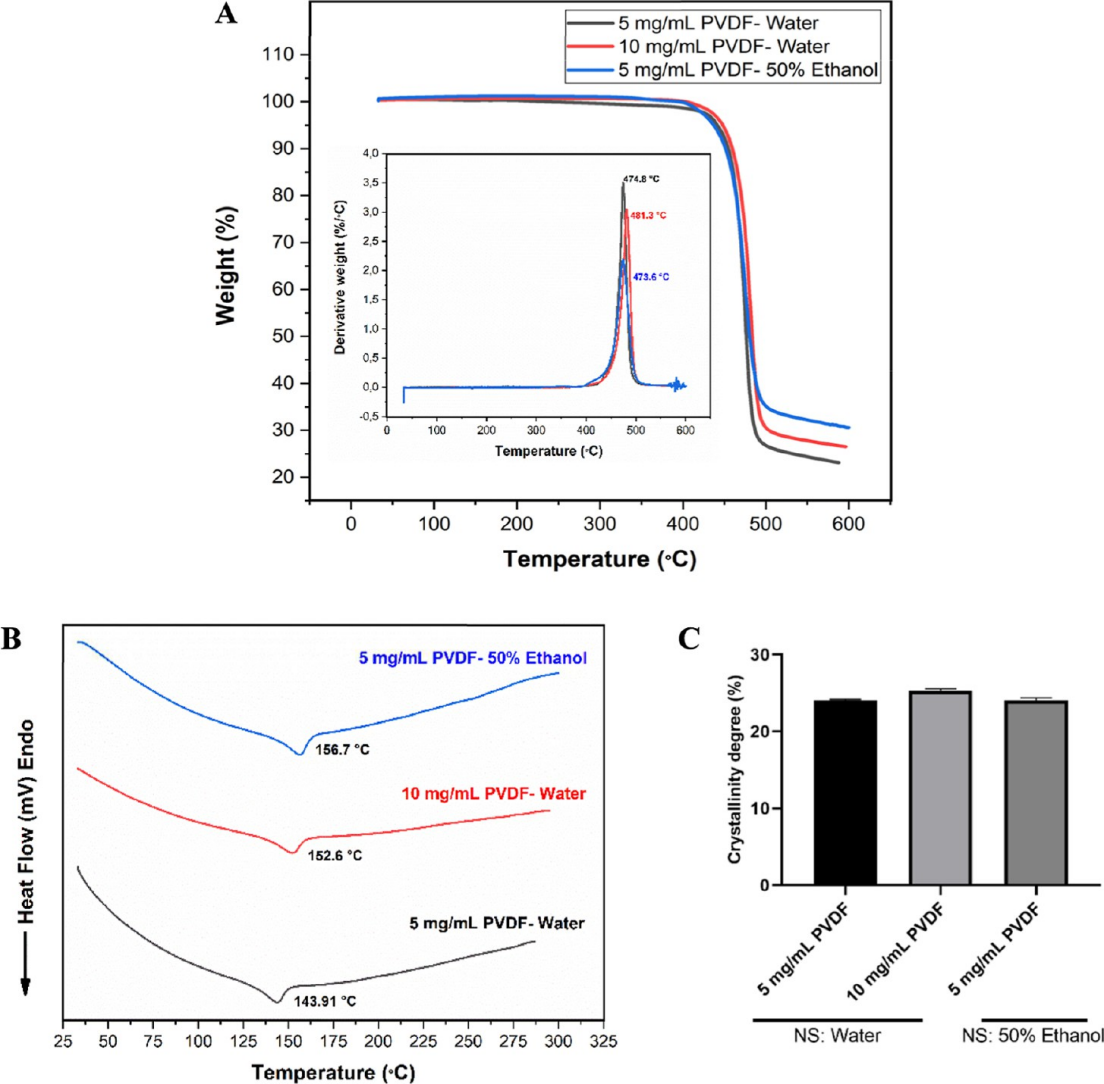
Thermal characterization of the PVDF nanoparticles: (A) TGA and corresponding DTG (inset) curves, (B) DSC curves, and (C) degree of crystallinity. NS represents the nonsolvent used.

There is an overlap of the thermogravimetric curves obtained, indicating no relevant differences in the thermal behavior of the nanoparticles (Figure 6A). Polymer degradation occurs in one step, mainly in the temperature range of 400–500 °C,^2^ as indicated by a single peak between 470 and 480 °C in the DTG curves. Therefore, the polymer particles showed good thermal resistance without deterioration after nanoparticle processing. However, the elimination of hydrogen fluoride and polymer-chain breakage, followed by the creation of char, suggests that degradation happens in two stages above that range of temperature.^30^ For example, the residual mass at 600 °C was between 20 and 35%.

DSC experiments on PVDF nanoparticles show a single endothermic peak between 143 and 157 °C (Figure 6B), which is associated with the melting point of the crystalline phases (α and β). However, it presents an offset in relation to the characteristic value of these phases (167–172 °C).^25^ In this case, the melting behavior of PVDF can be ascribed to the variation in morphology, possibly due to their nanometric conformation.^31^ It has already been widely reported in several mathematical models that the ratio between the surface and volume of nanoparticles affects the total energy and thermal stability of the system, which in turn affects the melting temperature, reducing it compared to their bulk counterparts.^32,33^ Lower structural order or the presence of defects in the nanoparticles contributes to melting at a lower temperature.^34,35^ According to these endothermic peaks, the degree of crystallization was also calculated using eq 2 and all particles presented values of approximately 25% (Figure 6C). Usually, PVDF films and membranes present higher values of crystallinity. Fernandes et al. reported that PVDF-based films present around 60% crystallinity and higher.^36,37^ Nevertheless, when they are reduced to nanometric sizes, many materials undergo a change in their crystalline structure or exhibit lower crystallinity. Such a moderate degree of crystallinity in PVDF nanoparticles can be beneficial when there is a need to encapsulate compounds. In fact, an optimal crystallinity degree for encapsulation is reported to be around 30%.^38^ Since PVDF is not biodegradable, a rigid crystalline structure could hinder the encapsulation process, making it more difficult for the encapsulate to be effectively incorporated. A lower degree of crystallinity allows for a more flexible structure, facilitating better encapsulation and release of the substances.

The consistent crystalline degree of PVDF nanoparticles across all developed samples, whether prepared with water or ethanol, may be attributed to the intrinsic crystallization properties of PVDF rather than the choice of nonsolvent. While water’s rapid solvent exchange may favor the formation of the electroactive β-phase and ethanol may result in a mix of phases, the overall crystalline degree is governed by thermodynamic equilibrium, ensuring similar extents of chain alignment and crystallization.^26,28,39^ The precipitation kinetics influenced by the nonsolvent impact the type of crystalline phase formed but not the total crystallinity.

### 3.4 Antimicrobial Activity Assessment: Static and Dynamic Conditions

The antimicrobial activity of the developed PVDF nanoparticles was tested under static and dynamic conditions using a Gram-positive *S. aureus* and a Gram-negative *E. coli*, with static conditions maintained at 0 rpm and dynamic conditions maintained at 200 rpm using a mechanical shaker for a duration of 2 h at 37 °C (Figure 7).

**Figure 7:**
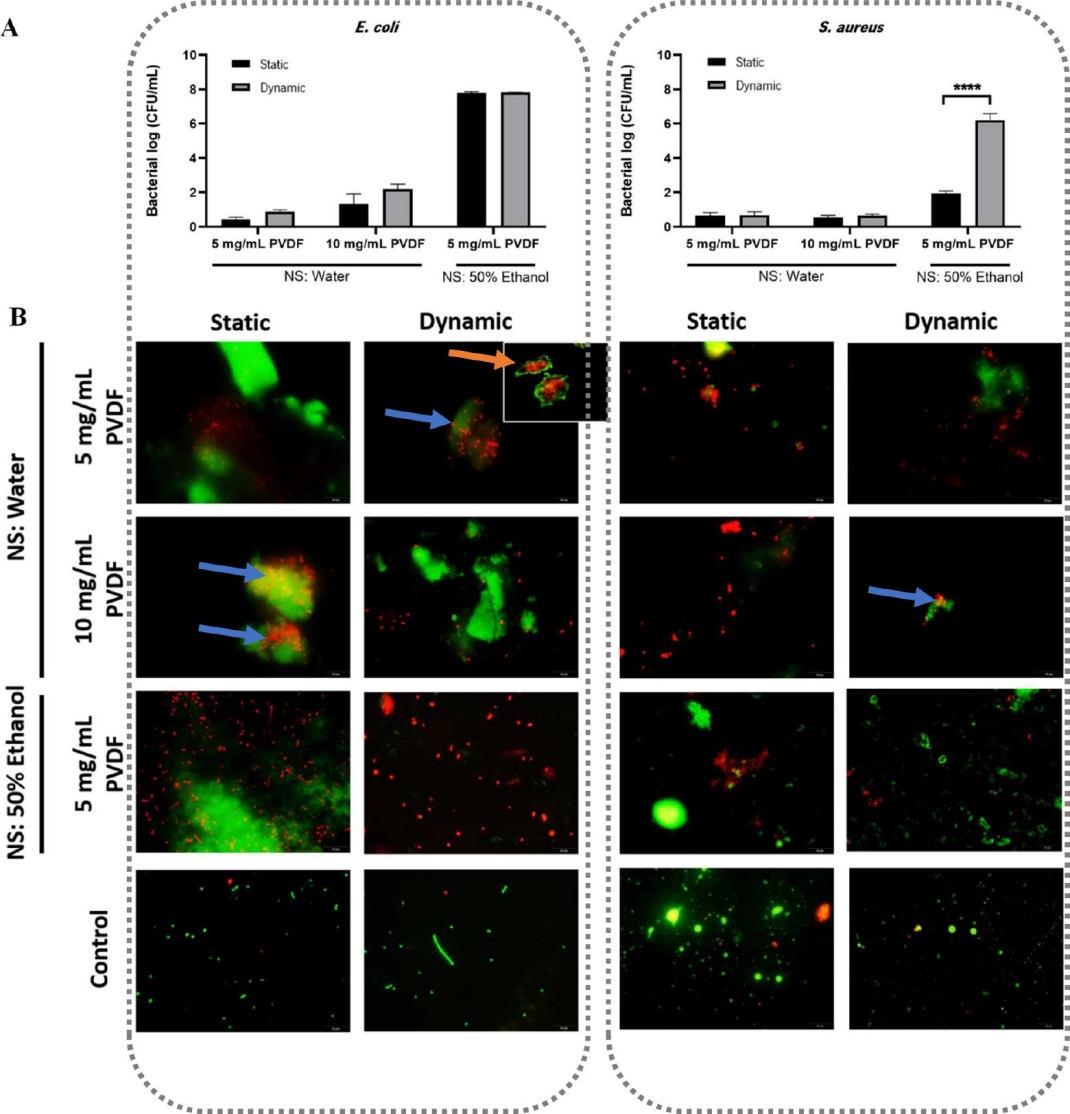
(A) Antibacterial activity as log_10_ (CFU/mL) of *E. coli* and *S. aureus* after 2 h incubation at 37 °C in contact with nanoparticles in static and dynamic conditions, the latter by applying a mechanical cue on the piezoelectric nanoparticles using 200 rpm. The results are the mean of two independent assays. ****P < 0.0001 static vs dynamic conditions. (B) Fluorescence microscopy live/dead images of *E. coli* and *S. aureus* after 2 h incubation at 37 °C in contact with nanoparticles in static and dynamic conditions. Live cells are represented in green and dead cells in red. However, the fluorescence of viable cells can be masked by the autofluorescence of PVDF, presented in the form of spots/clouds with green fluorescence. Scale bars indicate 10 μm for all images except the one highlighted with orange arrow (100 μm).

Observing Figure 7A, it is possible to depict that an inhibition of bacterial growth was observed, for both the static and dynamic conditions.

Under static conditions, even without mechanical movement, negatively charged PVDF nanoparticles, like other negatively charged nanoparticles, can induce contact killing through nonspecific binding and clustering at the cationic sites on the bacterial membrane.^5,20^ Furthermore, the size, composition, and surface characteristics of the material mainly determine its antibacterial effectiveness. The surface area of the material increases as the size decreases to the nanoscale, increasing the contact with microorganisms. Additionally, the aggregation of carbon nanostructures with bacterial cells leads to cell rupture.^40^ Still, in general, the dynamic condition seems to have a greater inhibitory effect and, in the case of *E. coli*, the effect is evidenced with the increase in PVDF concentration, with regard to particles whose nonsolvent used is water (for 10 mg/mL of PVDF there was a reduction of 1.33 log_10_ and 2.21 log_10_ in static and dynamic conditions, respectively). The same concentration effect is not as pronounced in *S. aureus* for the same particles.

In relation to particles whose nonsolvent used is a 50% ethanol solution, in both bacteria, there was a significant reduction. In the static condition, there was a full kill effect of the *E. coli* (7.80 log_10_ reduction) and a reduction of 1.93 log_10_ in the case of the *S. aureus*. However, once again, the dynamic condition presented a greater inhibitory effect, causing total death for both bacteria.

The results shown in Figure 7A can be justified by the combination of four effects: the negative charge of the particles causing death upon contact, the nanoscale of synthesized particles, the piezoelectricity of the PVDF nanoparticles, especially in the dynamic condition, and eventually the presence of ethanol in the particles, although part of it evaporated in the manufacturing process. For the piezoelectricity of the PVDF nanoparticles, studies have already proven that electrical microenvironments are generated through the stimulation of a piezoelectric polymer with a mechanical cue, thus developing an electrical response and a variation in the surface charge of the polymeric material, which can cause the inhibition of bacterial growth due to the rupture of its membrane.^6,41^ Furthermore, the significant differences between the two bacteria can be attributed to the structure of their membrane.^20^

Gram-negative bacteria have a thin layer of peptidoglycan (less than 10 nm) on their cell wall, which is adjacent to a cytoplasmic membrane and an outer membrane made of lipopolysaccharides. The positively charged parts of these membranes can interact with negatively charged particles and cause the membrane integrity to be compromised. The thick layer of peptidoglycan (20–80 nm) that makes up the membrane of Gram-positive bacteria, on the other hand, is composed of linear chains of polysaccharides that are cross-linked by short peptides. This gives the bacteria a negative charge from the upper network of the cell surface, which can repel the nanoparticles. This effect has already been reported in another study with negative-charged piezoelectric PVDF-based nanocomposites.^5^

In Figure 7B, live/dead fluorescent images of *E. coli* and *S. aureus* are shown, where the green fluorescent cells are viable while the red fluorescent cells have compromised cell membranes. However, the fluorescence of viable cells can be masked by the autofluorescence of PVDF.^40,42^ Fluorescence results corroborate those obtained using CFUs. In fact, there is a noticeable increase in bacterial death in *E. coli* compared to *S. aureus*, due to the number of nonviable cells depicted in Figure 7. Furthermore, a strong interaction seems to occur between the bacteria and the cluster of nanoparticles marked with green autofluorescence (highlighted by blue arrows), due to nonspecific binding and clustering of particles on cationic sites on the plasma membrane (that are relatively scarcer than negatively charged domains), as previously referred to.^20^ This phenomenon is even more evident in the case of the *E. coli* bacteria (highlighted by orange arrows), which may corroborate the interaction of the lipopolysaccharide’s membrane with the particles, compromising the integrity of the cell membrane.^5^

The interaction between the PVDF nanoparticles at a concentration of 10 mg/mL, synthesized without ethanol, and the bacteria was further studied in more detail using SEM, when subjected to dynamic conditions (200 rpm; Figure 8).

**Figure 8:**
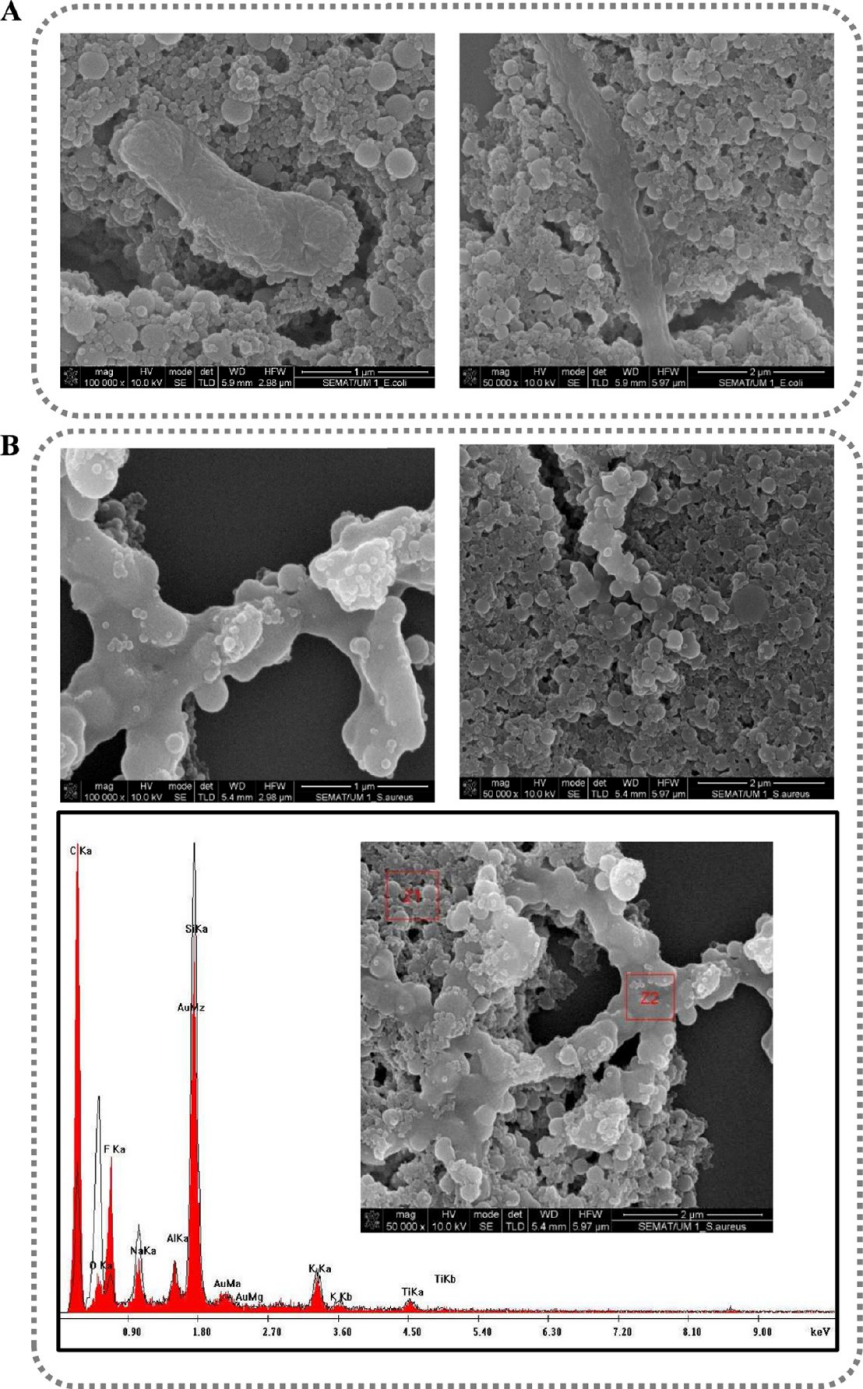
SEM images (100,000×, 50,000× magnification) of PVDF nanoparticles 10 mg/mL in contact with *E. coli* (A) and with *S. aureus* after dynamic conditions (200 rpm) (B). SEM-energy-dispersive spectrum (SEM-EDS) analysis of the nanoparticles (Z1—red spectrum) and *S.aureus* bacteria (Z2—black line spectrum) (B).

In fact, according to Figure 8, visible changes in the morphology of *E. coli* due to the interaction with PVDF nanoparticles are observed, inducing clear irregularities and alterations in its membrane that compromised the structural integrity of bacteria. Furthermore, these images also show a strong interaction and affinity between the nanoparticles and the surface of the bacteria, given that they are commonly found attached around the bacterial membrane. This interaction also occurs with the bacteria *S. aureus*, although it is much less evident. Less visible damage is observed and the cells appear more intact and smoother, reinforcing the hypothesis of electrostatic repulsion. Although some *S. aureus* bacteria are similar both visually and in terms of size when compared to the nanoparticles, their organization in a bunch-like structure allows them to be differentiated from the nanoparticles under study. Nevertheless, SEM-EDS analysis was performed in order to verify these differences. The SEM-EDS results show that in areas consisting of nanoparticles (Z1 red spectrum), there was an increase in carbon and fluorine and a reduction in oxygen since the PVDF polymer is composed of C, H, and F. Therefore, these results confirm the deposition of nanoparticles in these regions. On the other hand, in areas with predominance of *S. aureus* bacteria (Z2—black line spectrum), there was a reduction in carbon and fluorine, but an increase in oxygen, which is consistent with the composition of the bacterial cell wall, rich in polysaccharides and peptidoglycan (containing O, N, and less C). Furthermore, the fact that there is less fluoride and more oxygen in the areas where *S. aureus* bacteria predominate may also suggest that PVDF nanoparticles do not adhere strongly to the bacteria.

### 3.5 Cytotoxicity of PVDF Nanoparticles

When developing products intended for contact with human skin, it is essential to assess their potential cytotoxicity. Since the skin serves as the body’s primary barrier to external materials, evaluating the cytotoxicity of nanoparticles using human skin models is particularly critical. Epidermal fibroblasts are commonly used as an in vitro human skin model to assess the toxic effects of substances intended for skin contact. These cells play a key role in the process of cell renewal and in maintaining the integrity of the skin.^21^

The study of PVDF-based materials in the area of tissue engineering has already been highly reported. Studies with MC3T3-E1 preosteoblasts and human adipose stem cells with PVDF-based biomaterials for muscle tissue engineering have yielded highly promising results. Notably, the surface charge of the materials has been shown to significantly enhance cell proliferation and differentiation.^7^ Furthermore, studies have also demonstrated that PVDF combined with other polymers such as PU in the form of fibers for wound healing applications improves the activity/functionality of mouse embryo fibroblasts (both in vitro and in vivo).^43^ Another study on human fibroblasts proved that zeolite and clay PVDF-based composites are biocompatible materials that promote cellular response and do not display pro-inflammatory effects in vivo.^44^ However, the study of this material in its nanoparticle form has not yet been explored.

The potential cytotoxicity of PVDF-based particles was evaluated using an immortalized human skin fibroblast cell line (BJ-5ta). Figure 9 shows the metabolic viability of cells that grow directly in contact for 24 and 72 h with the nanoparticles and with the respective nonsolvents used for their manufacture. The cytotoxicity of the nonsolvent used was also evaluated to fully understand its contribution to the potential cytotoxicity of PVDF nanoparticles. To this end, BJ-5ta cells were exposed for 24 and 72 h to increasing dilutions of these nonsolvents that reach the same dilution that occurs with these solvents in the PVDF nanoparticles.

**Figure 9:**
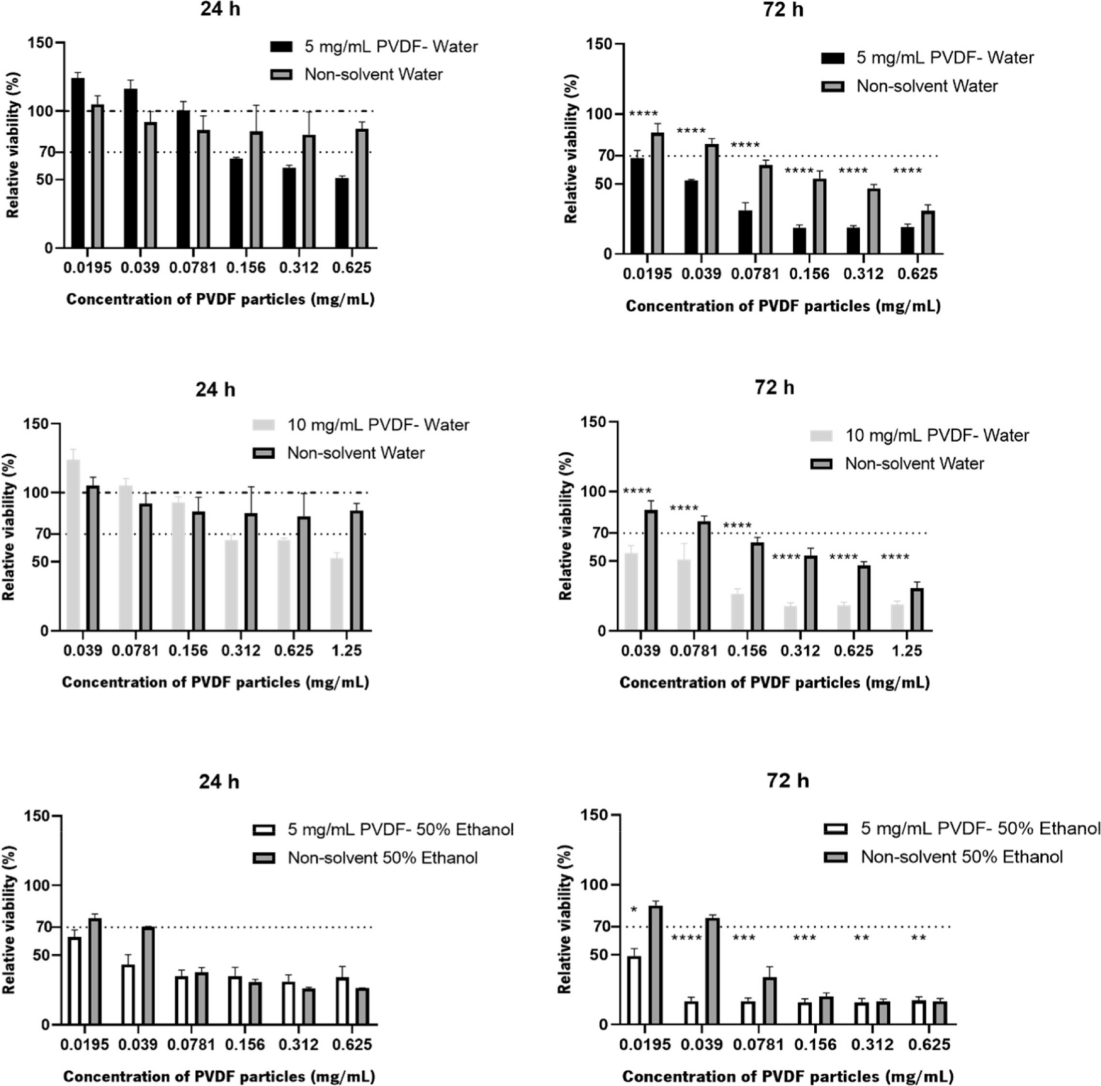
Relative viability of BJ-5ta human fibroblasts (evaluated by the MTS assay) after 24 and 72 h incubation with PVDF particles. Cells were exposed to different concentrations of PVDF particles in aqueous/ethanol suspension through successive 1:2 dilutions. The highest concentration tested for each PVDF nanoparticle suspension corresponds to the undiluted formulation. Data were calculated in relation to the live control—cells incubated only with culture media. Cells incubated with nonsolvent water or 50% ethanol at same dilutions as the particles were used as solvent controls. The data represent the mean ± SD from three independent experiments. Samples marked with ****, ***, **, and * show significative differences (****P-value ≤0.0001; ***P-value ≤0.001; **P-value ≤0.01; *P-value ≤0.1), when compared to the results obtained for the PVDF particles at 24 and 72 h, respectively.

At 24 h of incubation, the nonsolvent consisting only of water does not seem to induce a toxic effect for any of the dilutions tested, according to ISO 10328, as it showed cell viability greater than 70%. However, at 72 h, this solvent has already been shown to have a cytotoxic effect for almost all conditions, except for the two most diluted ones. This behavior may be derived from osmotic shock. Due to the variation in salt concentration between the surrounding solution and the interior of the cell, water can enter the cell, and it flows and subsequently explodes due to the fragile structure of the membrane of mammalian cells.^45^

Regarding particles prepared in this nonsolvent, a dose-dependent effect on cell viability was observed at 24 h of contact for both nanoparticles. When fibroblasts were exposed to increased concentrations of PVDF, cell viability was compromised at the highest concentrations of PVDF, with this effect being even more evident in 10 mg/mL PVDF particles than in 5 mg/mL PVDF particles. In fact, for particles made up of 5 mg/mL of PVDF, the three lowest concentrations did not show toxicity, adding a clear proliferative effect without influence of the nonsolvent used, since these concentrations showed cell viability greater than 100%. For nanoparticles made up of 10 mg/mL PVDF, this effect was also observed, but only for the two lowest concentrations. However, after 72 h of incubation, all PVDF particles prepared in water were cytotoxic regardless of the PVDF concentration, showing lower viability than that in water under the same conditions, meaning that the cell viability presented is mainly due to the nanoparticles themselves and not to the solvent used.

When the nonsolvent consisting of 50% ethanol solution was used, even if part of it evaporated in the nanoparticle synthesis process, some of this solvent may have remained in solution; therefore, the assessment of its toxicity alone is also crucial. In this case, both 24 and 72 h, it showed a cytotoxic effect for almost all conditions, except for the two most diluted, possibly due to ROS production.^46^ However, for the PVDF nanoparticles prepared in this ethanolic solution, both at 24 and 72 h, they showed a cytotoxic effect for all concentrations with even lower cell viability than the respective solvent, meaning that, similar to what happened with PVDF nanoparticles prepared in water after 72 h of contact with fibroblasts, the viability present here is suggestively due to the nanoparticles themselves and not to the nonsolvent used.

Therefore, comparing the viability results of the different nanoparticles, the particles whose nonsolvent used was a 50% ethanol solution were significantly more toxic than those prepared in water, in the first 24 h of incubation with the fibroblasts. Even so, all particles, regardless of the nonsolvent and PVDF concentration, showed cytotoxicity after 72 h of contact with fibroblasts.

## 4 Conclusions

This study focused on the development of PVDF-based nanoparticles, which exhibited excellent physical stability, nanometer-scale sizes, and low polydispersity. These characteristics underscore the PVDF polymer’s ability to crystallize and form uniform, smooth, spherical particles. The nanoparticles also demonstrated remarkable electroactivity, with a β-phase percentage exceeding 80%, and good thermal resistance, maintaining stability after processing. Additionally, they showed a crystallinity of approximately 30%, making them suitable for potential encapsulation applications. These properties represent a significant advancement in the area of materials science, demonstrating a synergistic effect between the antibacterial properties of PVDF negative nanoparticles and the applied piezoelectric stimulus. This combination effectively inhibits the growth of bacteria, particularly Gram-negative species like *E. coli*. The strong interaction between the nanoparticles and the bacterial membrane compromises membrane integrity, ultimately causing its rupture. The Gram-positive *S. aureus* exhibits greater resistance to this effect, which is attributed to the different structures of the cell membrane and its interaction with these nanoparticles. Furthermore, this study revealed that, within the first 24 h of incubation with fibroblasts, particles prepared using 50% ethanol as a nonsolvent solution are significantly more toxic than those prepared with water. However, all particles, regardless of the nonsolvent used or PVDF content, exhibited cytotoxicity after 72 h of interaction with fibroblasts. Despite this, the findings suggest that this antimicrobial strategy is both appropriate and promising for preventing bacterial infections, particularly on commonly touched public surfaces. By integrating these particles into composites or films, they can be utilized to create microbial-free coatings. The piezoelectric stimulus is naturally present in PVDF when crystallized in the β-phase, activated by movement, pressure, or touch, making it an ideal trigger for initiating antibacterial effects. Although the particles cannot be used in medical devices due to their toxicity, this study validates the mutually beneficial effects of a well-known antibacterial agent and an electroactive polymer in nanoform. These findings suggest that these particles, when combined with other materials, could serve as intelligent disinfectants that provide on-demand antimicrobial activity.
